# Solution-Grown Dendritic Pt-Based Ternary Nanostructures for Enhanced Oxygen Reduction Reaction Functionality

**DOI:** 10.3390/nano8070462

**Published:** 2018-06-26

**Authors:** Gerard M. Leteba, David R. G. Mitchell, Pieter B. J. Levecque, Candace I. Lang

**Affiliations:** 1 Department of Chemical Engineering, Catalysis Institute, University of Cape Town, Cape Town 7700, South Africa; pieter.levecque@uct.ac.za; 2 School of Engineering, Macquarie University, Sydney NSW 2109, Australia; 3 Electron Microscopy Centre, University of Wollongong, Wollongong NSW 2522, Australia; dmitchel@uow.edu.au

**Keywords:** thermal decomposition, ternary alloy, nanodendrites, surfactants, oxygen reduction reaction, electrocatalysts

## Abstract

Nanoalloys with anisotropic morphologies of branched and porous internal structures show great promise in many applications as high performance materials. Reported synthetic approaches for branched alloy nanostructures are, however, limited by the synthesis using a seed-growth process. Here, we demonstrate a conveniently fast and one-pot solution-phase thermal reduction strategy yielding nanoalloys of Pt with various solute feed ratios, exhibiting hyperbranched morphologies and good dispersity. When Pt was alloyed with transition metals (Ni, Co, Fe), we observed well-defined dendritic nanostructures in PtNi, PtCo and Pt(NiCo), but not in PtFe, Pt(FeNi) or Pt(FeCo) due to the steric hindrance of the trivalent Fe(acac)_3_ precursor used during synthesis. In the case of Pt-based nanoalloys containing Ni and Co, the dendritic morphological evolution observed was insensitive to large variations in solute concentration. The functionality of these nanoalloys towards the oxygen reduction reaction (ORR); however, was observed to be dependent on the composition, increasing with increasing solute content. Pt_3_(NiCo)_2_ exhibited superior catalytic activity, affording about a five- and 10-fold enhancement in area-specific and mass-specific catalytic activities, respectively, compared to the standard Pt/C nanocatalyst. This solution-based synthetic route offers a new approach for constructing dendritic Pt-based nanostructures with excellent product yield, monodispersity and high crystallinity.

## 1. Introduction

Improving the efficiency of catalysts for hydrogen proton exchange membrane fuel cells (PEMFCs) is an important challenge at present. Platinum is currently the catalyst of choice for PEMFCs [1,2,3,4], but the use of pure Pt is limited by its high cost. Pt also exhibits sluggish catalysis kinetics in the oxygen reduction reaction (ORR) [2,3,4,5].These factors constrain the commercial viability of PEMFCs and hence remain a barrier to their widespread use [2,6]. Considerable research is currently directed at improving the cost efficiency by synthesizing nanocatalysts with reduced Pt loading (achievable by alloying Pt with less-costly constituents) while also striving for accelerated ORR kinetics [1,2,3,4]. Nanoparticles with branched and porous structures exhibit improved catalytic activity owing to their exceptionally large surface area [5,7]. The design of more open structures in Pt-based nanoalloys has accordingly emerged as a promising platform for attaining improved catalytic performance [8]. There have been significant achievements in the design and preparation of more cost-effective Pt alloys than pure Pt, which exhibit excellent catalytic activity in the ORR: for instance, nanoalloys of Pt with Fe, Co or Ni [9,10,11,12]. Typically, however, these have been synthesized using seed-activated/-mediated growth followed by annealing; a synthesis protocol that poses scale-up challenges and requires prolonged annealing time [5,13,14,15,16].

Although nanoparticles with large surface area morphologies are expected to exhibit improved catalytic activity [5,7], the influence of variations in alloying composition on morphological evolution is less clear and requires systematic evaluation. Moreover, the scarcity of facile and direct one-pot synthetic approaches for rapid and large-scale production of alloy nanostructures with well-defined morphologies and controlled surface compositions required for industrial catalytic processes is indispensable. The correlation between the structure-composition-functionality relations of alloys is the driving force towards the design and development of new solution-phase synthetic approaches, leading to the manipulation of the size, composition, shape and structure of nanostructures. 

Herein, we report a conveniently fast and new one-pot high thermal (300 °C) decomposition approach, for the solution-phase synthesis of high quality dendritic nanostructures of Pt with varied stoichiometric solute feed ratios. We used this synthetic protocol to investigate the influence of composition on the morphology of a series of Pt nanoalloys. Assessment of morphology shows that this synthetic strategy (which does not require annealing) produces an open and branched morphology in Pt-based nanoalloys containing Ni and/or Co, but not Fe. Systematic variations in Ni and Co concentrations were not observed to result in morphological changes to these nanoalloys, which have a large surface area, porous internal structure and many low coordination sites at edges and corners [5,17,18]. The catalytic activity is however sensitive to composition, increasing with decreasing Pt content (i.e., with increasing Ni or Co concentration) and hence decreasing cost.

## 2. Experimental Methods

### 2.1. Synthesis of Binary PtNi, PtCo and PtFe Nanostructures

In a typical thermolysis synthesis: Metal precursor salts Pt(acac)_2_ (0.2 g, 0.5085 mmol), with Ni(Ac)_2_ (0.12 g, 0.4822 mmol), Co(Ac)_2_ or Fe(acac)_3_ (0.17 g, 0.4822 mmol), were dissolved by stirring in oleylamine (OAm, 20 mL), trioctylamine (TOA, 15 mL) and oleic acid (OLEA, 5 mL) at 200 °C for 10–15 min, in a high boiling point solvent, 1-octadecene (1-ODE, 25 mL). Thereafter, the resultant homogeneous solution was rapidly transferred into a one-neck flask and heated to 300 °C for 15–20 min, with a heating rate of 15 °C/min. Upon completion, the reaction mixture was allowed to cool to room temperature, and the colloidal particles were flocculated by the addition of excess ethanol and acetone and washed three times to ensure the elimination of any unwanted solvent and excess surfactants. The black product was dried and finally re-suspended in chloroform (20 mL) by mild sonication, yielding a dark-brown homogeneous colloidal suspension.

### 2.2. Synthesis of Ternary Pt(NiCo), Pt(FeNi) and Pt(FeCo) Nanostructures

In a typical thermolysis synthesis: Pt(acac)_2_ (0.2 g, 0.5085 mmol), with (Ni(Ac)_2_ (0.06 g, 0.2411 mmol) + Co(Ac)_2_ (0.06 g, 0.2411 mmol), or (Fe(acac)_3_, (0.085 g, 0.2411 mmol) + Ni(Ac)_2_ (0.06 g, 0.2411 mmol), or (Fe(acac)_3_ (0.085 g, 0.2411 mmol) + Co(Ac)_2_ (0.06 g, 0.2411 mmol), were dissolved by stirring in OAm (20 mL), TOA (15 mL) and OLEA (5 mL) at 200 °C for 10–15 min, in 1-ODE (25 mL). Thereafter, the resultant homogeneous solution was rapidly transferred into a one-neck flask and heated to 300 °C for 15–20 min.

Under the same synthesis conditions, Pt_2.64_(NiCo)_2_ and Pt_3.5_(NiCo)_2_ were solution grown by decreasing the NiCo stoichiometric feed ratios as follows: Ni(Ac)_2_ (0.048 g, 0.1929 mmol) + Co(Ac)_2_ (0.048 g, 0.1929 mmol) and Ni(Ac)_2_ (0.036 g, 0.1447 mmol)+ Co(Ac)_2_ (0.036 g, 0.1447 mmol), respectively.

### 2.3. Nanostructure Characterization Techniques

The black powders of the as-synthesized and unsupported nanostructures were deposited onto a silicon (Si) wafer support and characterized by powder X-ray diffraction (XRD) on an X’Pert Pro multipurpose diffractometer (MPD), using Cu Kα radiation (λ = 1.54056 Å). The XRD patterns were recorded at a scan rate of 0.106°/s and with a step size of 0.0334°. Scanning transmission electron microscopy (STEM) specimens were prepared by placing one drop of the colloidal solution (nanoparticles re-dispersed in chloroform) on 3-mm copper grids coated with a carbon support. The grids were dried under ambient conditions. Thereafter, the materials were analysed using transmission electron microscopy (TEM), high-resolution TEM (HR-TEM) and STEM on a JEOL ARM200F (JEOL, Tokyo, Japan) probe-corrected instrument, operating at 200 keV. The chemical compositions of individual nanostructures were determined using energy dispersive X-ray spectroscopy (EDS) in STEM mode. Spectrum imaging was used in which an EDS spectrum was obtained at each pixel in the STEM Image, to produce a 3 dimensional (3D) dataset. Rapid acquisition was used (5 s per frame) integrated over at least 100 frames. Image drift correction was applied after each frame. The 3D datasets were analysed with the Noran System Seven software. STEM imaging was carried out in high angle annular dark field (HAADF), bright field (BF) and secondary electron (SE) modes. Fourier transform infrared (FT-IR) spectra of the as-synthesized alloy nanostructures physically mixed with KBr pellets were acquired using a Nicolet 5700 FT-IR spectrometer (Thermo Fisher Scientific, Madison, WI, USA).

### 2.4. Electrochemical Measurements

Cyclic voltammetry (CV) scans were conducted in an argon (Ar)-purged electrolyte by potential cycling of the working electrode between 0.05 V and 1.00 V vs. the standard hydrogen electrode (SHE) at a scan rate of 100 mV/s in a 0.1 M HClO_4_ solution. The electrocatalysts were observed to stabilize after 100 voltage cycles, which were used to electrochemically clean the catalyst surface, referred to as “catalyst activation” [19]. The sweep rate was then reduced to 50 mV/s, and the third cycle at that scan rate was used for analysis. The electrochemically-active surface area (ECSA) was calculated by integrating the area under the curve for the hydrogen underpotential deposition region (H_upd_) assuming a monolayer hydrogen charge of 210 µC/cm^2^_Pt_ [20,21]. Carbon monoxide (CO)-stripping voltammetry curves were obtained by bubbling CO gas into the electrolyte solution while holding the potential of the working electrode (WE) at 0.1 V vs. SHE. The electrolyte was then purged with Ar to remove the dissolved CO gas while still holding the potential of the WE at 0.1 V vs. SHE. The potential of the WE was then cycled to 1.00 V vs. SHE at 20 mV/s, followed by a CV cycle as described above at 20 mV/s. The peak area could then be determined using the baseline CV, and a normalization factor of 420 µg/cm^2^_Pt_ [22] was used to calculate the ECSA_CO_. For the linear sweep voltammetry (LSV) curves, the potential of the WE was swept from 1.10 V to 0.20 V vs. SHE and back at 10 mV/s. ORR polarization curves were recorded at rotation speeds of 1600 rpm. The ORR curves obtained in oxygen (O_2_)-saturated electrolyte were corrected for the capacitive current associated with Pt*_x_*M*_y_*/C catalysts, by subtracting a CV measured in an Ar-saturated electrolyte. The current densities were also normalized with reference to the calculated ECSA to evaluate the specific activities. For polarization curves, the measured currents were corrected for mass transport to acquire the true kinetic currents. The mass activities and specific activity were determined at +0.9 V by normalizing the kinetic currents (I_k_) with the ECSA of the alloy catalysts and the Pt catalyst, respectively, immobilized on the electrode. The kinetic current (I_k_) can be calculated by using the Koutecky–Levich equation [23].

## 3. Results and Discussion

### 3.1. Influence of Different Metal Precursors on Nucleation and Growth Mechanism of Pt 3D Transition Metals (Ni, Co, Fe)

**Figure 1:**
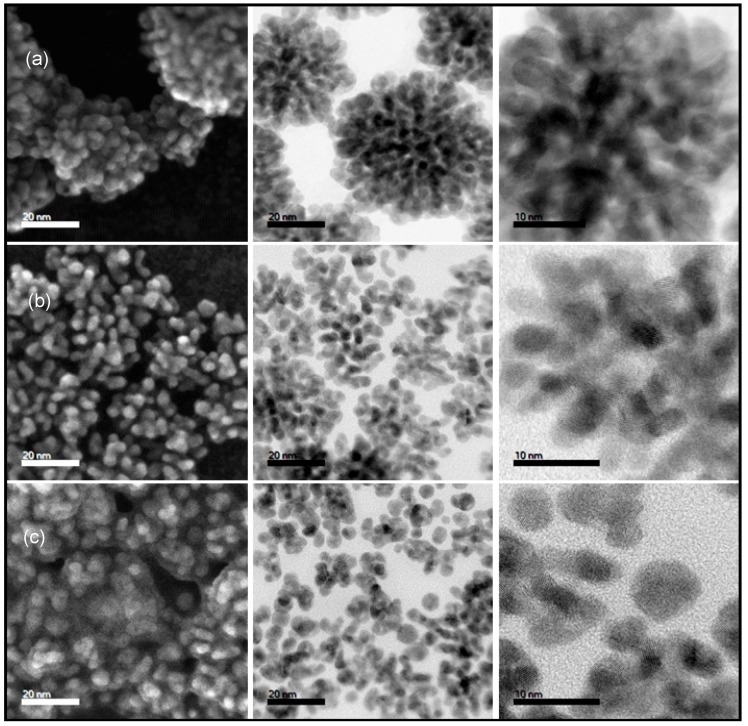
Left to right, STEM images corresponding to: secondary electron (SE), bright field (BF) and high resolution BF images of ternary Pt-based nanoalloys: (**a**) Pt(NiCo); (**b**) Pt(FeNi); and (**c**) Pt(FeCo).

The size and morphological evolution of solution-grown metallic nanostructures are governed by nucleation and growth kinetics, which can be controlled by experimental parameters including the nature of precursor salts, reduction temperature, reducing agents and surfactants [17,18,24,25]. We accordingly explored a high temperature (300 °C) co-thermal decomposition approach to balance complete decomposition of the metal precursors into zero valent states, in the presence of distinct hydrophobic surfactants of different functional groups: oleylamine (OAm), trioctylamine (TOA) and oleic acid (OLEA). Figure 1 (left to right, respectively) shows scanning transmission electron microscopy (STEM) images corresponding to secondary electron (SE), bright-field (BF) images and high-resolution BF images of three ternary nanoalloys synthesized with different solutes. Pt(NiCo) nanoalloys (Figure 1a) are observed to form well-defined and monodisperse dendritic morphologies; whereas Pt(FeNi) (Figure 1b) and Pt(FeCo) (Figure 1c) show a mixture of segregated spherical and interconnected nanoalloys. The presence of Fe in Pt(FeNi) or Pt(FeCo) nanoalloys is accordingly associated with irregular morphologies, unlike the highly dendritic structure of Pt(NiCo) nanoalloys. Binary nanoalloys of Pt with Co, Ni or Fe were synthesized using the same thermolysis protocol. Images show monodisperse nanostructures of PtCo and PtNi with dendritic morphology; PtFe exhibited a mixture of polydispersed single-crystalline and polycrystalline nanostructures ranging from spherical, tripod, tetrapod and irregular morphologies (Supplementary Materials Figure S1). The simultaneous coexistence of both single-crystalline and polycrystalline nanostructures has previously been observed [25,26]. These results, together with the results presented in Figure 1, show that for this high temperature decomposition synthetic protocol, the Co(Ac)_2_ and Ni(Ac)_2_ metal salts are associated with the development of a uniform dendritic morphology.

We interpret the observed morphological evolution differences in terms of the different oxidation states of divalent Ni^2+^ and Co^2+^ cations_,_ compared to the trivalent Fe^3+^ cation. Ni and Co precursor salts possess a single short ligand, which cleaves off efficiently during reduction; as a consequence, the growing crystal sites permit more incorporation of incoming zero valent Ni^0^ and Co^0^. In the case of Fe(acac)_3_ with three large ligands, we hypothesise that the cleaving of the ligand bonds occurs in a stepwise manner when Fe binds to the growing nanoparticles. This in turn generates cluster-Fe(acac)_2_ group as an intermediate. These large, dangling ligands sterically block the incorporation of similar Fe-containing molecules and also those containing Pt, Ni or Co. It thus appears that trivalent Fe(acac)_3_ modifies the growth behaviour of the Fe-containing nanoalloys due to steric hindrance. This, in turn, suggests that the reduction kinetics of the Fe(acac)_3_ precursor affects the nucleation and subsequent crystal overgrowth of nanostructures. This results in irregular morphologies for the Fe-containing nanoalloys, in which symmetrical branch formation and the development of highly interconnected patterns of crystal facets are inhibited.

### 3.2. The Influence of Alloying Feed Ratios on the Morphology of Pt(NiCo) Alloys

**Figure 2:**
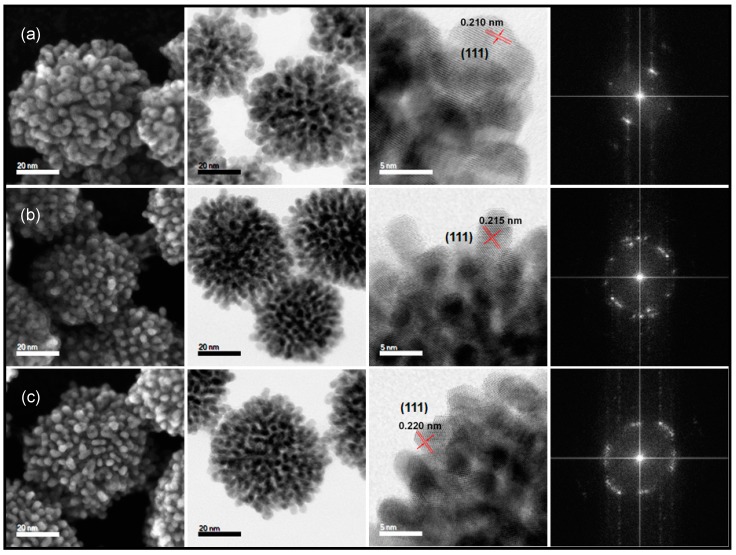
STEM images corresponding to (left to right): secondary electron (SE), bright field (BF), high resolution (HR) BF and FFT diffractograms (generated from the HR-BF images) of the as-synthesized ternary Pt-based nanoalloys with precursor compositions (**a**) Pt_3_(NiCo)_2_; (**b**) Pt_4_(NiCo); and (**c**) Pt_5_(NiCo). Based on HR-STEM images ((**a**,**b**,**c**), third column), the measured *d*-spacings are 0.210 nm, 0.215 nm and 0.220 nm, respectively.

In Figure 2 are shown (left to right) STEM SE, BF, HR-BF and the corresponding fast Fourier transform (FFT) diffractograms of ternary nanostructures solution-grown by the synthetic protocol described, while systematically varying the feed ratio between Pt:(NiCo) precursor salts as follows: 2:(1:1), 2.6:(1:1), and 3.5:(1:1). All nanostructures are observed to have a dendritic morphology that radiates out from the core; and a high surface area exhibiting multiple crystal facets. The STEM images show that varying the feed ratio of solute precursors had a negligible effect on the final morphology of Pt(NiCo) nanoalloys. The average particle diameters of these three ternary nanostructures (calculated from the measurement of approximately 300 individual nanoparticles) was 63 nm–73 nm, with broader, skewed particle size distributions (Supplementary Materials Figure S2). HR-BF images reveal polycrystalline nanoalloys with well-resolved lattice fringes (the measured *d*-spacings are shown on the figures) and randomly-oriented crystal facets. The multiplicity of facets arises from the deposition of single crystals, from the core outward, which results in the evolution of multiply-exposed crystal facets. This is further confirmed by the FFT diffractograms, which show arcs of spots characteristic of polycrystalline structures, in contrast with disparate spot patterns identified when imaging separate single or twinned crystals. This shows that nanocrystals exhibit a narrow range of orientations, suggesting that templating has occurred: the orientation of new crystals is guided by the growth of pre-existing surfaces. Thus, our high temperature co-reduction approach suggests that the crystallographic origin of these selectively monodisperse polycrystalline dendritic nanostructures, regardless of composition variations, is due to the rapid growth of preformed individual alloy nanocrystals, coalescing at favourably-oriented/attachment sites rather than guided or accompanied by epitaxial growth. It is, however, possible that the deployment of ternary surfactants can alter the growth kinetics and dictate the nanostructure growth orientation because of preferential binding or nonbinding on growing crystals, thus favouring coalescence and aggregation-directed growth of preformed single crystals in a diffusion-controlled manner [7].

**Figure 3:**
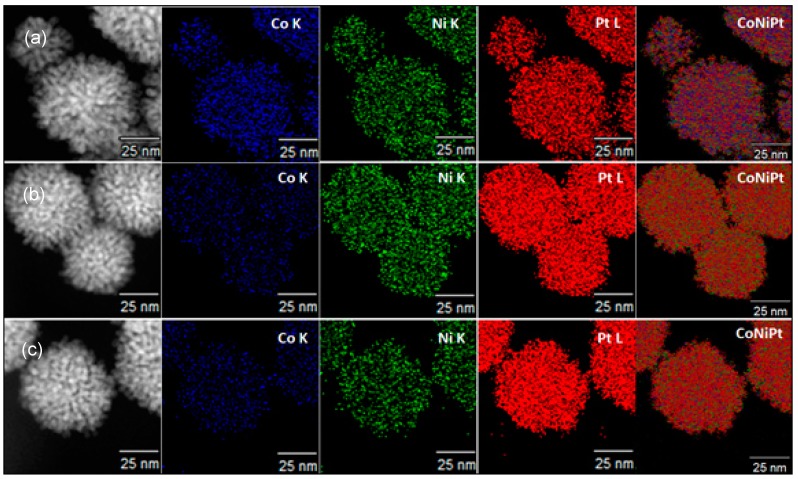

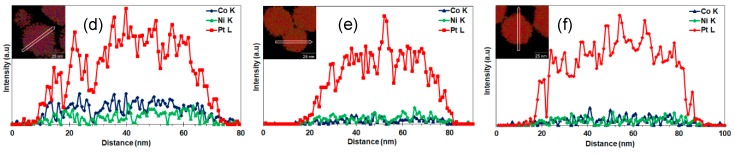
HAADF-STEM-EDS elemental mapping results for ternary alloys synthesized as (**a**) Pt_3_(NiCo)_2_, (**b**) Pt_4_(NiCo) and (**c**) Pt_5_(NiCo), exhibiting a homogeneously mixed distribution of Co, Ni and Pt within the nanoalloys. The blue, green and red colours in the HAADF-STEM images represent Co, Ni and Pt, respectively. (**d**–**f**) show line scan profiles through regions in **a**-**c** respectively along the lines (white) shown in the inserts.

We next evaluated the distribution of elements within the Pt(NiCo) nanoalloys, as shown in Figure 3. A homogeneous atomic distribution of Pt and the alloying elements is observed for all nanoalloys. The average atomic compositions of the ternary nanoalloys were determined by energy dispersive spectroscopy (EDS) to be Pt_60_(NiCo)_40_, Pt_79_(NiCo)_21_ and Pt_82_(NiCo)_18_ (Supplementary Materials Figure S3). The intensity profiles acquired through elemental maps of the composite images in Figure 3a–c reveal an even distribution of the three elements across single nanoparticles. The crystal structure of ternary Pt(NiCo) nanoalloys was evaluated by X-ray diffraction (XRD) (Supplementary Materials Figure S4). Five 2θ diffraction peaks were indexed to the (111), (200), (220), (311) and (222) planes, characteristic of a face-centred cubic (fcc) solid solution. There were no additional XRD peaks detected of pure Pt and (NiCo), indicating that the fcc phase is a single-phase disordered solid solution. A slight shift of the peak positions toward higher angles as solute content increases, relative to pure Pt, suggests a decreased lattice parameter, consistent with the replacement of some Pt by smaller atoms of Ni and Co in the crystal lattice [27,28]. In the case of dendritic binary nanostructures (Supplementary Materials Figure S5), XRD measurements (Supplementary Materials Figure S6) show PtCo to have a larger lattice parameter than PtNi, as expected from the relative atom size of Co and Ni. Additionally, these binary nanoalloys displayed typical diffraction peaks that could be indexed to an fcc solid solution. The average particle diameters (calculated from the measurement of approximately 300 individual nanoparticles) of binary PtNi and PtCo were 73 ± 8.8 nm and 63 ± 5.4 nm, respectively, exhibiting broader and skewed particle size distributions (Supplementary Materials Figure S7). Further EDS compositional and HAADF-STEM-EDS elemental mapping assessments of the as-synthesized binary Pt-based nanostructures gave average atomic compositions of Pt_52_Ni_48_ and Pt_54_Co_46_ (Supplementary Materials Figures S8 and S9, respectively), consistent with the 1:1 feed ratio (of precursor salts) used during synthesis. Based on the results obtained, the systematic compositional variations and analysis indicate that hyperbranched nanostructures with more accessible surface and porous internal structures can be solution-grown using the described co-thermal synthetic approach.

### 3.3. The Effect of Synthesis Chemistry on Nanoalloys’ Morphological Evolution

The thermolysis method in this work aimed to provide a rapid synthesis medium, which resulted in dendritic Pt nanoalloys within 15–20 min, hence a shortened reaction period. The growth mechanism of pure Pt into dendritic structures is reported to occur anisotropically along the <111> orientation, leading to the formation of interconnected branches at low temperatures (≤150 °C) and spherical Pt structures at the elevated temperature (250 °C), using OAm as both surfactant and reductant [26]. This may suggest that the OAm molecules are tightly bound to the crystal surfaces at lower temperatures and dictate the final morphology of the particles. However, this synthesis required prolonged annealing time for the complete formation of such dendritic nanostructures [26,29]. Our experiments suggest that the evolution and growth of dendritic nanostructures is rapid at 300 °C, stimulating fast coalescence/stacking of single crystals in a highly controlled interconnected manner. In addition, these nanostructures were observed to sediment during synthesis, yielding a dense black product. We correlate this phase transformation mechanism of crystallization/precipitation to induced weakened binding of the surfactants and supersaturation of single crystals in a synthesis solution phase at the elevated temperature. Generally, single crystals tend to grow into larger particles, to minimize the interfacial energy and consequently yielding dense colloids with diminished surface energy or may trigger subsequent particle coarsening (Ostwald ripening) [24], with prolonged reaction time. In the case of polycrystalline NPs, total atomic diffusion or anisotropic particle-particle coalescence can be hindered by crystalline boundary/lattice diffusion, thereby favouring the construction of interconnected NPs with internal pores and high surface free energy [18]. By decreasing the stoichiometric feed ratios of the alloying elements, we observed that the dendritic morphological evolution remains unaltered. Furthermore, this implies that Pt anisotropic growth could be the key determinant in the creation of such branched and interconnected nanostructures. Our results are, however, contradictory to the creation of spherical nanocrystals at the elevated temperature [26]. 

In this work, dendritic nanostructures were observed for Pt with Ni and/or Co, but not Fe. Accordingly, we consistently used the same Pt, Co and Ni precursor salts and decomposition temperature (300 °C) together with OAm, TOA and OLEA to achieve simultaneous reduction of the metal salts and hence nanoalloy nucleation (note that the three surfactants act also as reductants here). Subsequent growth of the nanoalloys was controlled by the selection of surfactants (OAm, TOA and OLEA) to promote the growth of a dendritic nanostructure. Surfactants passivate and coat the developing nanostructures during growth: the adsorption of stabilizers on surfaces differs in strength depending on the orientation of surface facets. This directs the rate at which surfactant monomers attach to different surface facets [30]. The binding affinity (adsorption) of two or more surfactants on growing crystal surfaces within the same wet synthetic system differs. The surfactants tightly bound on crystal surfaces provide more steric hindrance, arresting the rate of crystal growth and thereby providing an intimate organic coating shell in the overall final particle growth. The weakly-bound stabilizers serve for on/off surface attachment/detachment, accelerating growth [31]. These weakly-bound organic molecules are washed off facilely during the flocculation/purification (cleaning) process of the as-synthesized nanostructures.

**Figure 4:**
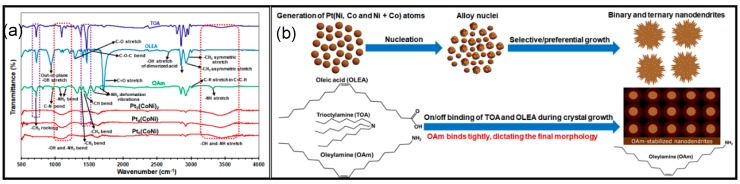
(**a**) FT-IR spectra of surfactants oleylamine (OAm), oleic acid (OLEA), trioctylamine (TOA) and highly-branched nanostructures of Pt_3_(NiCo)_2_, Pt_4_(NiCo) and Pt_5_(NiCo); (**b**) schematic illustration of the proposed crystal growth mechanism of both the binary and ternary nanostructures of Pt with Ni and/or Co, synthesized in the presence of a homogeneous mixture of three surfactants.

In order to elucidate this metal-surface functionalization/competition of ternary organic surfactants (OAm, OLEA and TOA), Fourier transform infrared (FTIR) measurements (Figure 4a) of the three distinct surfactants and the as-synthesized ternary nanostructures were conducted. The recorded spectra of OAm, OLEA and TOA are in good agreement with other reports [28,32]. Accordingly, the ternary nanostructures’ recorded spectra are similar to that of OAm, but not OLEA and TOA. The pure OLEA absorption bands (not observed in the spectra of TOA, OAm and ternary alloys) around ~1712 cm^−1^ and ~2680 cm^−1^ are characteristic of the carboxylic group (C=O) stretching mode and hydroxyl group (–OH) stretching mode, respectively, of the dimerized acid [33,34]. Pure OAm exhibits typical bands at ~1562 cm^−1^ and ~1650 cm^−1^ (not appearing in either TOA or OLEA spectra) attributed to the NH_2_ deformation vibrations/scissoring mode of primary amines, whereas the peak at ~3328 cm^−1^ is assigned to the –NH stretching mode [33]. The OAm absorption bands appear in the spectra of all ternary nanostructures. These FT-IR spectral investigations gathered suggest that the organic coordinating agents attached on the as-synthesized ternary nanostructures after repeated destabilization/purification approach are exclusively OAm, although both OLEA and TOA were used during solution-grown alloys, in conjunction with OAm.

We deduce that the organic surfactants determining the dendritic morphological evolution/transformation of these ternary nanoalloys are OAm, as schematically illustrated in Figure 4b. Multiple surfactants used in the same synthetic system can thus trigger preferential adsorption in a range of surface orientations, leading to distinct crystallographic growth directions and hence to a dendritic structure. Our synthetic approach resulted in the successful formation of high surface area, multiply-branched nanoalloys Pt(NiCo) with varied stoichiometric compositions. These highly-exposed different crystal facets and porous internal structures may foster active reaction sites, thus enhancing the functionality of the binary and ternary alloys depending on the surface compositions. 

### 3.4. Catalyst Activity Measurements towards ORR

**Figure 5:**
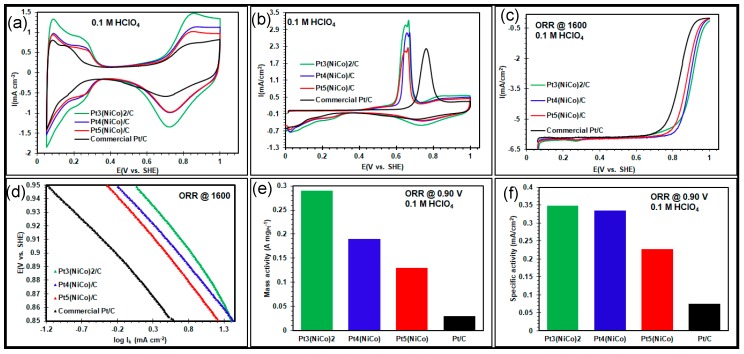
Pt_3_(NiCo)_2_/C, Pt_4_(NiCo)/C, Pt_5_(NiCo)/C and commercial Pt/C electrocatalysts: (**a**) cyclic voltammograms; (**b**) CO-stripping voltammetry; (**c**) ORR polarization curves; (**d**) the corresponding Tafel plots; (**e**) mass-specific activities; and (**f**) intrinsic area-specific activities at +0.9 V.

Prior to electrochemical evaluations, dendritic Pt(NiCo) nanoalloys of varying composition were first deposited on carbon black (Vulcan XC-72R, Fuel Cell Store, Austin, TX, USA) via a colloidal-deposition approach. TEM images of these particles showed no apparent change in particle size or morphology following dispersion onto the support (Supplementary Materials Figure S10). The surface electrochemical properties of these ternary nanoalloys were then measured and compared to commercial Pt/C (HiSPEC 60% on carbon). Figure 5a shows cyclic voltammetry (CV) curves for the four nanocatalysts after voltage cycling to 100 cycles of the catalyst’s surface cleaning. These curves exhibit both the hydrogen desorption/adsorption peak (at 0.05–0.35 V) and the oxide formation/reduction peak (at 0.7–1.0 V) [35,36]. The magnitude of both of these peaks (in mA.cm^−2^) scaled in the following sequence: Pt_3_(NiCo)_2_/C > Pt_4_(NiCo)/C > Pt_5_(NiCo)/C > Pt/C. The calculated ECSA_Hupd_ (in m^2^g_Pt_^−1^) scaled in the same sequence: Pt_3_(NiCo)_2_/C (78.5) > Pt_4_(NiCo)/C (57.2) > Pt_5_(NiCo)/C (52.7) > Pt/C (38.8). The latter is of interest because it shows that the nanoalloys with the highest solute content also have the highest ECSA_Hupd_, although the morphology of all nanoalloys is dendritic. Figure 5b shows the CO-stripping voltammetry curves. Sharp, well-defined transient positive current CO peaks were observed for the three nanoalloys, between +0.55 and +0.75 V. The ECSA_CO_ (in m^2^g_Pt_^−1^) scaled as follows: Pt/C (85.1) > Pt_3_(NiCo)_2_/C (80.1) > Pt_4_(NiCo)/C (63.6) > Pt_5_(NiCo)/C (54.7). This is consistent with the ECSA_Hupd_ for ternary nanoalloys, but not for Pt/C. 

The ECSA_CO_/ECSA_Hupd_ ratio for all the ternary nanoalloys was 1.02–1.11, indicating little difference between H_ads_ and CO_ads_ surface coverage. In contrast, the ECSA_CO_/ECSA_Hupd_ ratio for the commercial Pt/C electrocatalyst was ~2.2, which suggests that CO is better adsorbed on Pt surfaces than on the ternary nanoalloy surfaces. This can be explained by the smaller average sizes of the Pt nanocatalysts (7.3 ± 4.2 nm) and higher Pt coverage (60 wt %) on the carbon surface. Figure 5b also shows that the presence of Ni and Co on the Pt nanoalloy surfaces is associated with a shift in the CO stripping peaks to lower potentials than the commercial Pt/C nanocatalyst. This suggests that the presence of Ni and Co improves CO tolerance [37]. Double CO oxidation peaks were also observed for ternary alloys. A number of factors can give rise to this phenomenon in these alloys, including: the existence of defects; segregated particles [38]; preferential/selective binding onto distinct facets [39]; the nature of the surface sites or particle size distribution [38,40]. 

The influence of composition on the ORR functionality was probed in an O_2_-purged 0.1 M HClO_4_ electrolyte solution at room temperature. There are two observable potential regimes in the ORR polarization curves in Figure 5c: a mixed, kinetic-diffusion-controlled region (the true measure of the catalyst functionality) between 0.8 and 1.0 V and a diffusion-limited current density regime between ~0.1 and 0.8 V. In the latter region, all the catalysts reached the theoretical limiting current density of −6.02 mA/cm^2^ [23,41,42]. The Tafel plots shown in Figure 5d, obtained from the potentials of 0.85–0.95 V, exhibit functionalities that scaled as follows: Pt_3_(NiCo)_2_/C > Pt_4_(NiCo)/C > Pt_5_(NiCo)/C > Pt/C. These plots thus show that Pt_3_(NiCo)_2_/C displays the highest positive kinetic currents of all the electrocatalysts at any given potential, suggesting exceptional catalytic performance of ternary nanoalloys of this composition. Figure 5e shows the calculated Pt mass-specific activity (A mg_Pt_^−1^) that scaled as follows: Pt_3_(NiCo)_2_/C (0.29) > Pt_4_(NiCo)/C (0.19) > Pt_5_(NiCo)/C (0.13) > Pt/C (0.03). The Pt nanoalloys thus showed mass-specific activity up to 10-times higher than Pt/C. Figure 5f shows that the area-specific activities (in mA.cm^−2^) scale in the same sequence, Pt_3_(NiCo)_2_/C (0.35) > Pt_4_(NiCo)/C (0.34) > Pt_5_(NiCo)/C (0.23) > Pt/C (0.075), showing the Pt nanoalloys to have values up to five-times that of Pt/C. These outstanding ECSAs and the corresponding ORR functionalities are attributed to the alloying composition and the associated open nature of their morphology [8,43,44,45].

## 4. Conclusions

We demonstrate a rapid thermolysis protocol (requiring less annealing time) to synthesise high-quality Pt-based nanoalloys with hyperbranched morphologies. We have determined that, using this fast and low-cost protocol, Pt nanoalloys with Ni and/or Co solute exhibit a dendritic morphology, not observed in Pt nanoalloys containing Fe. Furthermore, in these Pt nanoalloys with Ni and Co, the morphology remains dendritic when the host:solute ratio is varied. Their open-framework morphology confers a high surface area, which allows significant molecular accessibility to surface atoms. All Pt(CoNi) nanoalloys display outstanding catalytic functionality for the sluggish ORR: our electrochemical measurements show that these nanoalloys exhibit functionality enhancement in both mass-specific and area-specific activities compared to the state-of-the-art commercial Pt/C catalyst. The catalytic activity of these nanoalloys is observed to increase with increasing solute concentration, offering both a cost advantage and a catalytic advantage, relative to standard Pt catalysts. Although our preliminary data on the electrochemical measurements conducted suggest that these high surface area ternary Pt(NiCo) nanoalloys display enhanced ORR functionality, they were observed to degrade under corrosive environments even after 100 potential cycles of catalyst surface cleaning. This observation is in line with the research findings by Cui et al. where the structural transformation of active nanostructures resulted in diminished activity with prolonged potential cycling [19]. The accelerated durability tests (ADTs) of these dendritic ternary nanostructures thus require further investigations. The synthesis effort reported here provides a new opportunity for further development of cost-effective Pt-substituted nanostructures as high-performance electrocatalysts.

## Supplementary Materials

The following are available online at http://www.mdpi.com/2079-4991/8/7/462/s1: Figures S1–S10 (PDF).
