# Supplementary material for: Solution-Grown Dendritic Pt-Based Ternary Nanostructures for Enhanced Oxygen Reduction Reaction Functionality
Source: Nanomaterials (Basel). 2018 Jun 26;8(7):462. doi: 10.3390/nano8070462 (PMC6070889; doi:10.3390/nano8070462)
Supplement: Supplementary file 1 [file nanomaterials-08-00462-s001.pdf]

Supporting Information for

Solution-Grown Dendritic Pt-Based Ternary Nanostructures for Enhanced Oxygen Reduction Reaction Functionality

Gerard M. Leteba[1,2], David R.G. Mitchell[3], Pieter B.J. Levecque[1] and Candace I. Lang[2*]

[1] Catalysis Institute, Department of Chemical Engineering, University of Cape Town, South Africa
[2] School of Engineering, Macquarie University, Australia
[3] Electron Microscopy Centre, University of Wollongong, Australia
* Correspondence: gerard.leteba@uct.ac.za, candace.lang@mq.edu.au; Tel.: +61 9850 9149

Figures S1–S9

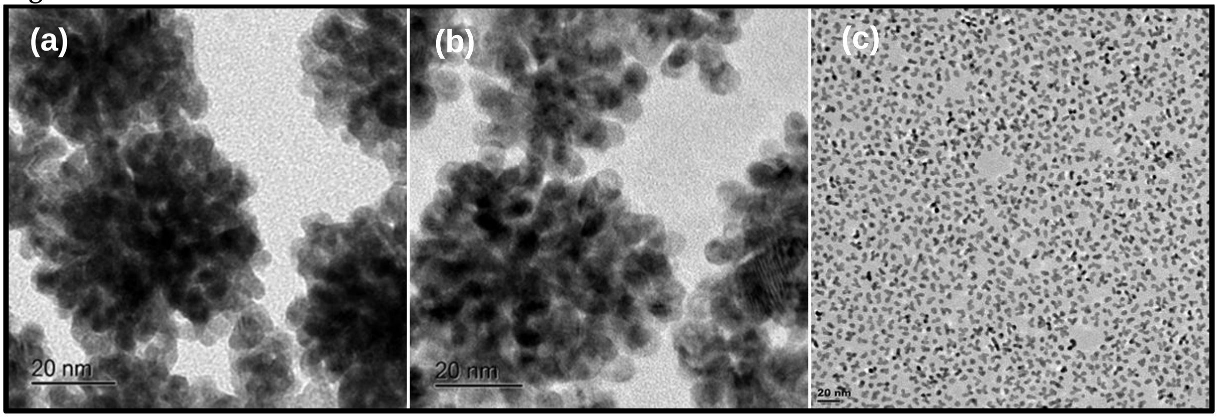

Figure S1. Bright field STEM micrographs of binary Pt-based nanostructures, synthesized using 1:1 feed ratios: (a) PtCo, (b) PtNi and (c) PtFe.

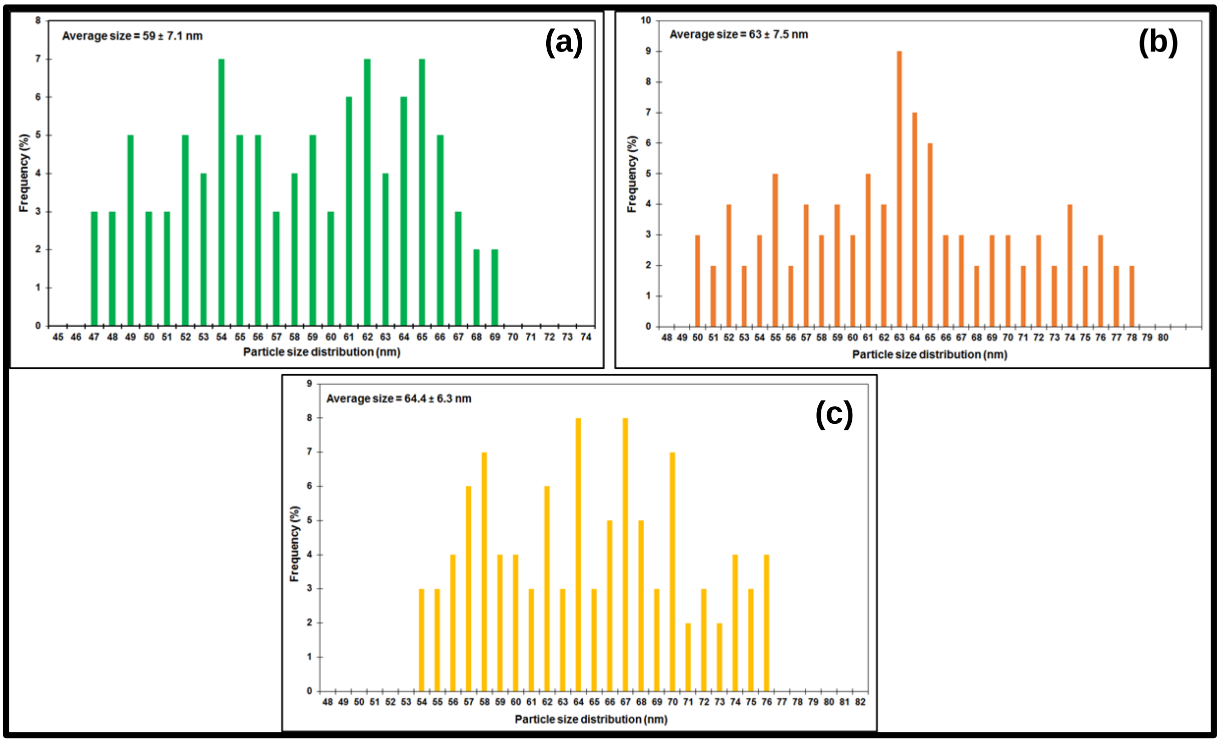

Figure S2. Particle size (nm) histograms of (**a**) $Pt_3(NiCo)_2$, (**b**) $Pt_4(NiCo)$ and (**c**) $Pt_5(NiCo)$ alloy nanostructures.

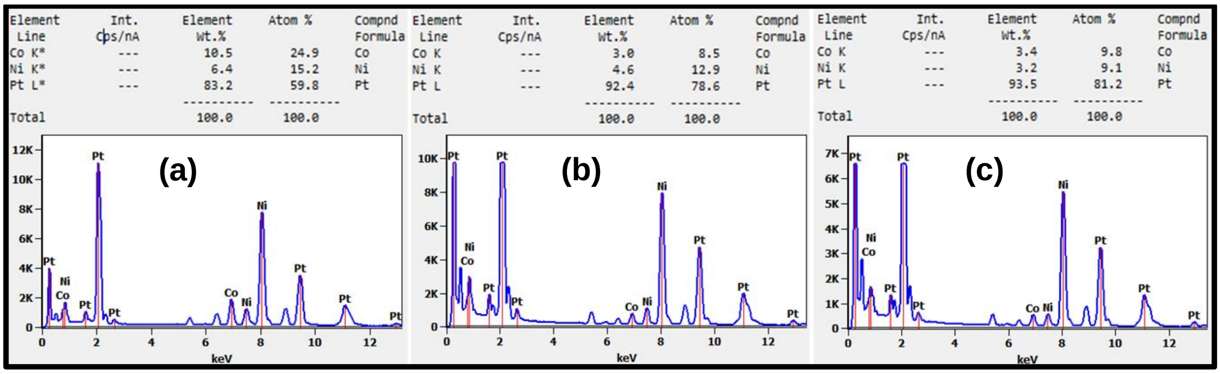

Figure S3. EDX spectra and elemental compositions of (**a**) $Pt_3(NiCo)_2$, (**b**) $Pt_4(NiCo)$ and (**c**) $Pt_5(NiCo)$ ternary alloy nanostructures.

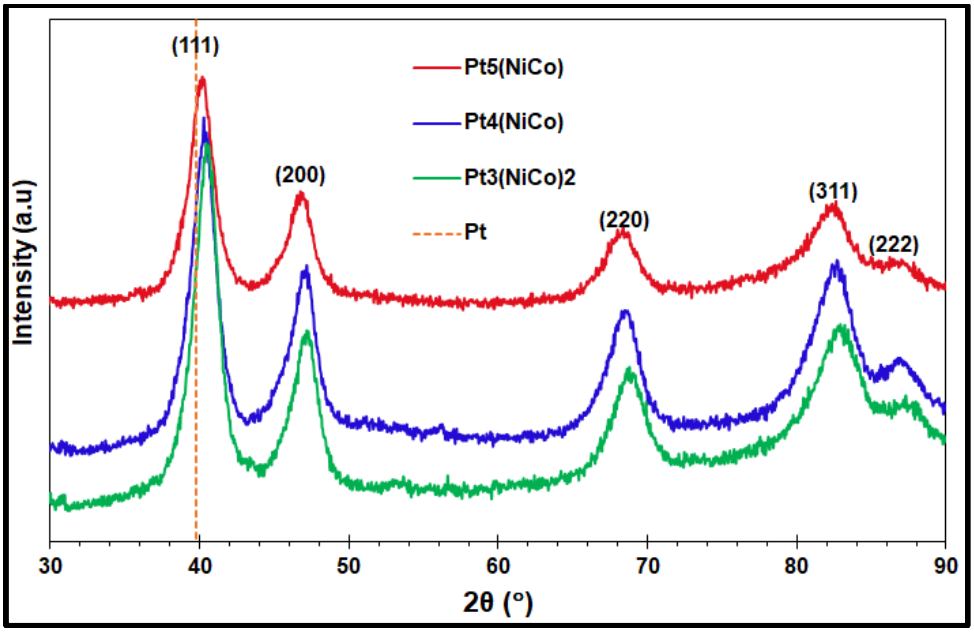

Figure S4. XRD patterns of $Pt_3(NiCo)_2$, $Pt_4(NiCo)$ and $Pt_5(NiCo)$ nanoalloys. The {111} peak position of pure Pt is indicated.

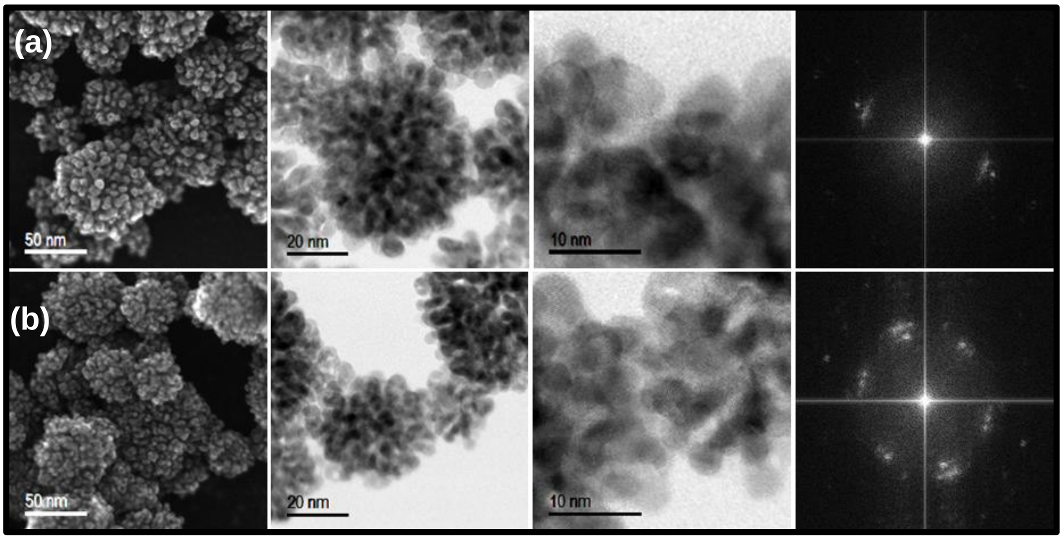

Figure S5. SEM (SE) images, BF-STEM images, HR-STEM images and the corresponding FFT patterns of binary (**a**) PtNi and (**b**) PtCo nanostructures.

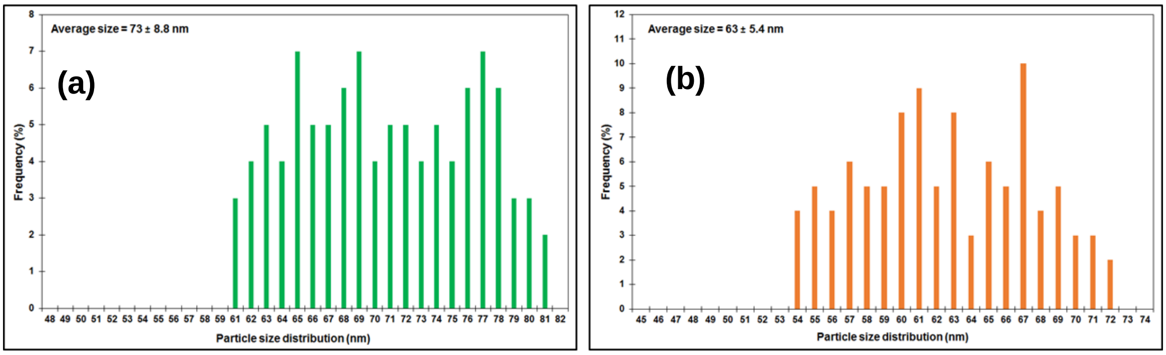

Figure S6. Particle size (nm) histograms of (**a**) PtNi and (**b**) PtCo nanostructures.

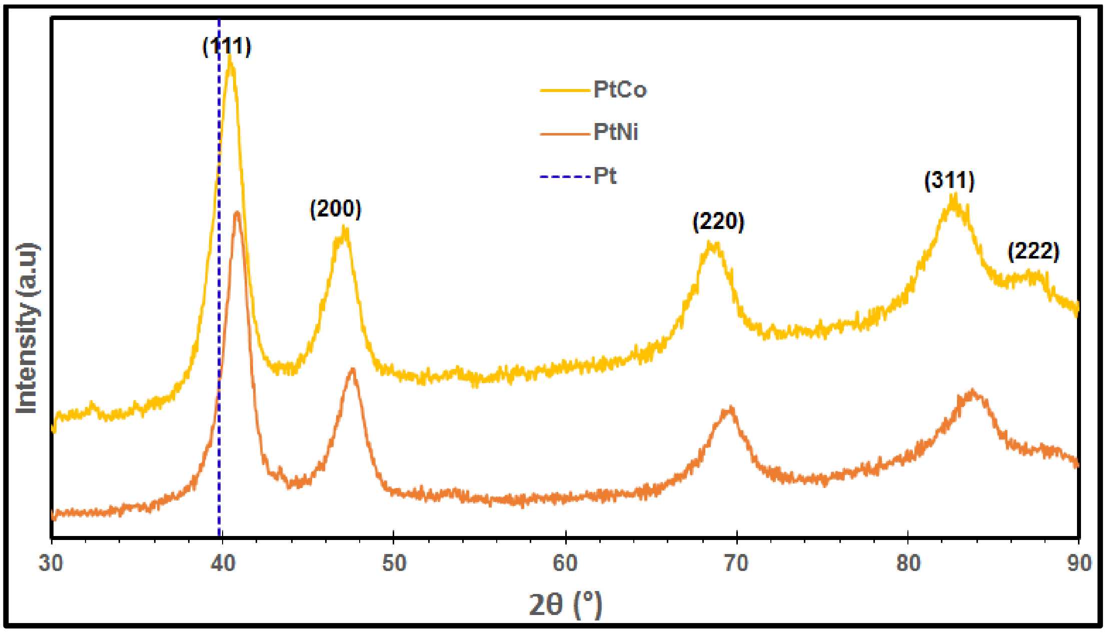

Figure S7. XRD patterns of binary PtCo and PtNi alloy nanoparticles. The position of the Pt(111) peak is shown as a dotted line.

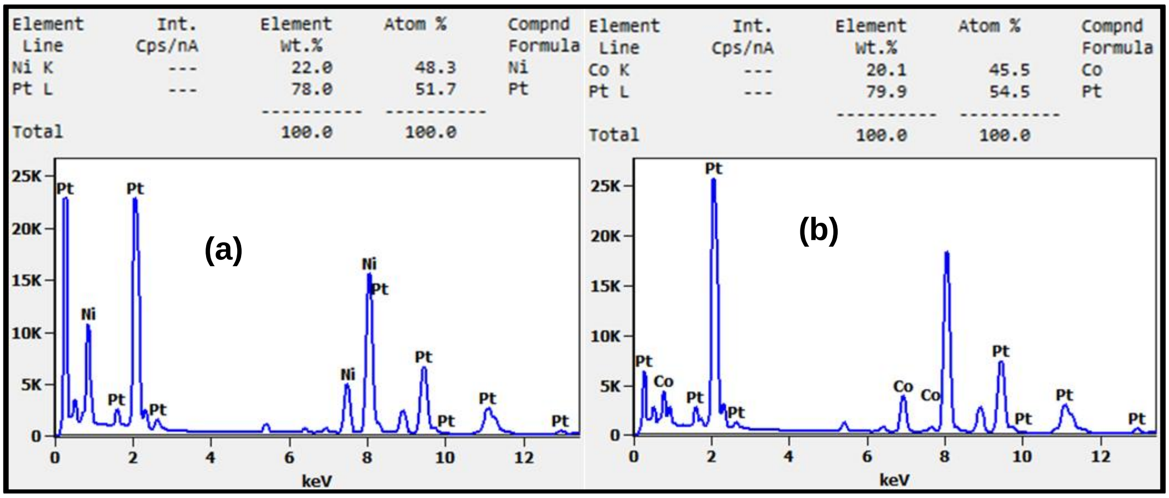

Figure S8. EDX spectra and elemental compositions of (**a**) PtNi and (**b**) PtCo binay alloy nanostructures.

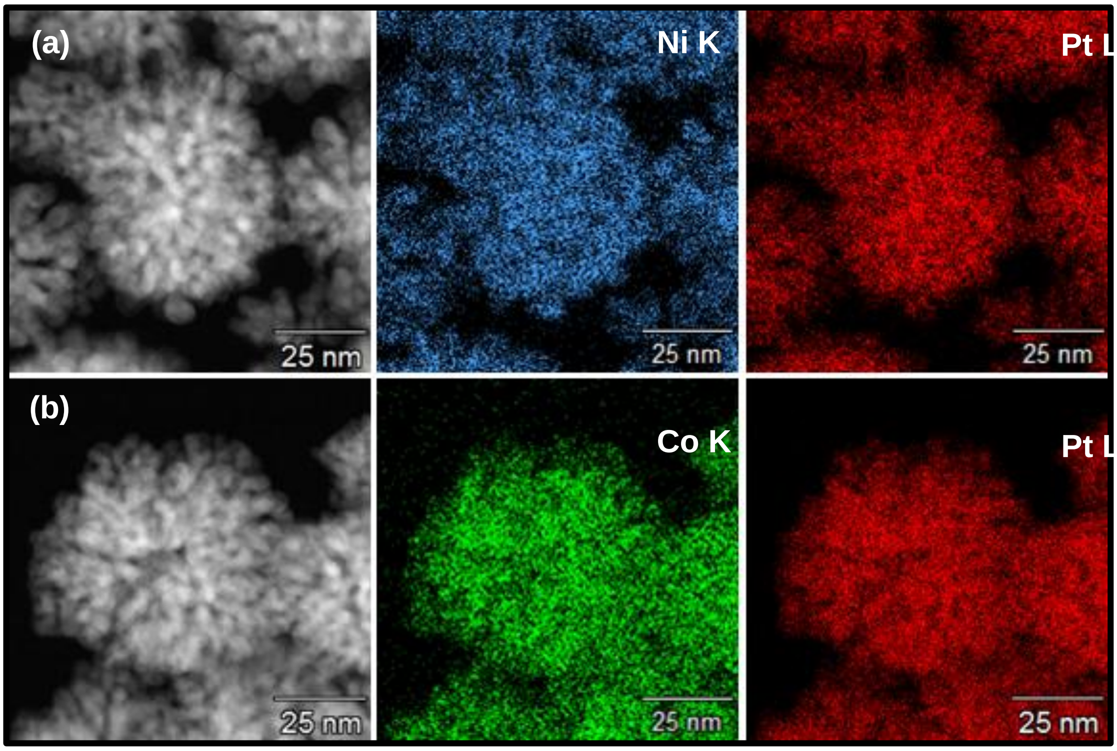

Figure S9. HAADF-STEM-EDS analysis: nanoscale elemental mapping of (**a**) PtNi and (**b**) PtCo nanostructures, showing uniform atomic distributions of alloying elements (Ni or Co) and Pt within individual particles.

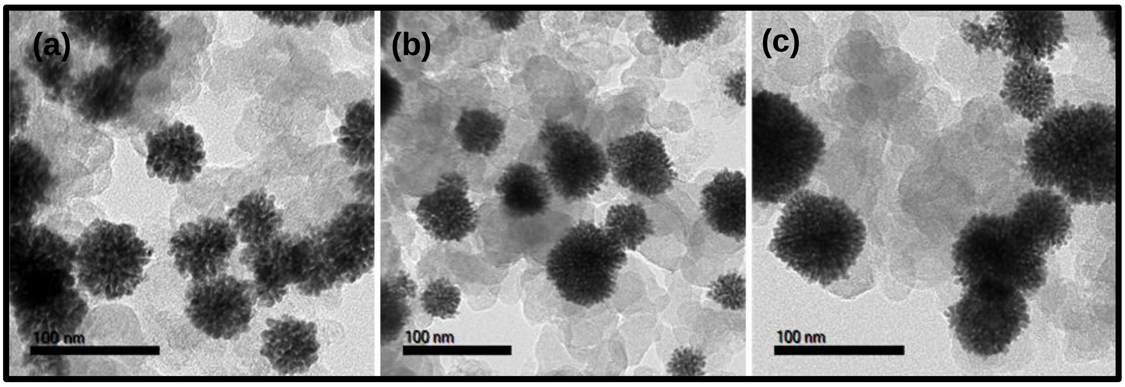

Figure S10. TEM images of (**a**) $Pt_3(NiCo)_2$, (**b**) $Pt_4(NiCo)$ and (**c**) $Pt_5(NiCo)$ nanoalloys, dispersed on high surface area carbon (Vulcan XC-72R).
